# Supplementary material for: Impact of stress and coping strategies on quality of life in hematological malignancies: A cross-sectional study
Source: PLoS One. 2025 Sep 8;20(9):e0331865. doi: 10.1371/journal.pone.0331865 (PMC12416831; doi:10.1371/journal.pone.0331865)
Supplement: S1 Table — (DOCX) [file pone.0331865.s001.docx]

S1 Table.

STROBE Statement—checklist of items that should be included in reports of observational studies

|  | | Item No | Recommendation | | Reported on page |
| --- | --- | --- | --- | --- | --- |
| **Title and abstract** | | 1 | (*a*) Indicate the study’s design with a commonly used term in the title or the abstract | | 1-2 |
|  |  |  | (*b*) Provide in the abstract an informative and balanced summary of what was done and what was found | | 3 |
| Introduction | | | | |  |
| Background/rationale | | 2 | Explain the scientific background and rationale for the investigation being reported | | 3-4 |
| Objectives | | 3 | State specific objectives, including any prespecified hypotheses | | 5 |
| Methods | | | | |  |
| Study design | | 4 | Present key elements of study design early in the paper | | 5 |
| Setting | | 5 | Describe the setting, locations, and relevant dates, including periods of recruitment, exposure, follow-up, and data collection | | 5 |
| Participants | | 6 | (*a*) *Cohort study*—Give the eligibility criteria, and the sources and methods of selection of participants. Describe methods of follow-up  *Case-control study*—Give the eligibility criteria, and the sources and methods of case ascertainment and control selection. Give the rationale for the choice of cases and controls  *Cross-sectional study*—Give the eligibility criteria, and the sources and methods of selection of participants | | 6 |
|  |  |  | (*b*) *Cohort study*—For matched studies, give matching criteria and number of exposed and unexposed  *Case-control study*—For matched studies, give matching criteria and the number of controls per case | |  |
| Variables | | 7 | Clearly define all outcomes, exposures, predictors, potential confounders, and effect modifiers. Give diagnostic criteria, if applicable | | 7 |
| Data sources/ measurement | | 8* | For each variable of interest, give sources of data and details of methods of assessment (measurement). Describe comparability of assessment methods if there is more than one group | | *7-9* |
| Bias | | 9 | Describe any efforts to address potential sources of bias | | 5 |
| Study size | | 10 | Explain how the study size was arrived at | | 9 |
| Quantitative variables | | 11 | Explain how quantitative variables were handled in the analyses. If applicable, describe which groupings were chosen and why | | 9 |
| Statistical methods | | 12 | (*a*) Describe all statistical methods, including those used to control for confounding | | 9 |
|  |  |  | (*b*) Describe any methods used to examine subgroups and interactions | | 9 |
|  |  |  | (*c*) Explain how missing data were addressed | |  |
|  |  |  | (*d*) *Cohort study*—If applicable, explain how loss to follow-up was addressed  *Case-control study*—If applicable, explain how matching of cases and controls was addressed  *Cross-sectional study*—If applicable, describe analytical methods taking account of sampling strategy | |  |
|  |  |  | (*e*) Describe any sensitivity analyses | |  |
|  | |  |  | |  |
| Results | | | | |  |
| Participants | 13* | | | (a) Report numbers of individuals at each stage of study—eg numbers potentially eligible, examined for eligibility, confirmed eligible, included in the study, completing follow-up, and analysed | 10 |
|  |  |  |  | (b) Give reasons for non-participation at each stage | 10 |
|  |  |  |  | (c) Consider use of a flow diagram |  |
| Descriptive data | 14* | | | (a) Give characteristics of study participants (eg demographic, clinical, social) and information on exposures and potential confounders | 10 |
|  |  |  |  | (b) Indicate number of participants with missing data for each variable of interest |  |
|  |  |  |  | (c) *Cohort study*—Summarise follow-up time (eg, average and total amount) |  |
| Outcome data | 15* | | | *Cohort study*—Report numbers of outcome events or summary measures over time |  |
|  |  |  |  | *Case-control study—*Report numbers in each exposure category, or summary measures of exposure |  |
|  |  |  |  | *Cross-sectional study—*Report numbers of outcome events or summary measures | *12* |
| Main results | 16 | | | (*a*) Give unadjusted estimates and, if applicable, confounder-adjusted estimates and their precision (eg, 95% confidence interval). Make clear which confounders were adjusted for and why they were included | 15 |
|  |  |  |  | (*b*) Report category boundaries when continuous variables were categorized |  |
|  |  |  |  | (*c*) If relevant, consider translating estimates of relative risk into absolute risk for a meaningful time period |  |
| Other analyses | 17 | | | Report other analyses done—eg analyses of subgroups and interactions, and sensitivity analyses | 16-18 |
| Discussion | | | | |  |
| Key results | 18 | | | Summarise key results with reference to study objectives | 19 |
| Limitations | 19 | | | Discuss limitations of the study, taking into account sources of potential bias or imprecision. Discuss both direction and magnitude of any potential bias | 23 |
| Interpretation | 20 | | | Give a cautious overall interpretation of results considering objectives, limitations, multiplicity of analyses, results from similar studies, and other relevant evidence | 20-22 |
| Generalisability | 21 | | | Discuss the generalisability (external validity) of the study results | 24 |
| Other information | | | | |  |
| Funding | 22 | | | Give the source of funding and the role of the funders for the present study and, if applicable, for the original study on which the present article is based |  |
