# Supplementary material for: Impact of stress and coping strategies on quality of life in hematological malignancies: A cross-sectional study
Source: PLoS One. 2025 Sep 8;20(9):e0331865. doi: 10.1371/journal.pone.0331865 (PMC12416831; doi:10.1371/journal.pone.0331865)
Supplement: S2 Table — Note: Overall QoL: R2 = 0.365, Adj. R2 = 0.327, F = 9.611, p < 0.001. Functional Fields: R2 = 0.456, Adj. R2 = 0.423, F = 13.975, p < 0.001. Symptomatic Fields: R2 = 0.342, Adj. R2 = 0.302, F = 8.664, p < 0.001. (DOCX) [file pone.0331865.s002.docx]

S2 Table. The 95% confidence intervals (CIs) of all variables in the models

| Multiple linear regression analysis of overall life quality in patients with hematological diseases. | | | | | | |
| --- | --- | --- | --- | --- | --- | --- |
| **Variables** | **Standard**  **error** | **Standardized regression coefficient** | **t-value** | **P-value** | **95% Confidence Interval** | |
|  |  |  |  |  | **Lower Limit** | **Upper Limit** |
| Constant | 17.047 |  | 4.211 | 0.000 | 38.135 | 105.445 |
| Sex | 3.215 | 0.137 | 2.135 | 0.034 | 0.518 | 13.215 |
| Age | 3.211 | -0.094 | -1.459 | 0.146 | -11.027 | 1.653 |
| Marital status | 1.972 | 0.115 | 1.798 | 0.074 | -0.348 | 7.441 |
| Place of Residence | 1.344 | -0.138 | -1.93 | 0.055 | -5.249 | 0.059 |
| Type of insurance | 2.869 | 0.066 | 0.935 | 0.351 | -2.981 | 8.346 |
| Crisis perception | 0.494 | -0.223 | -2.859 | 0.005 | -2.386 | -0.437 |
| Coping ability | 0.739 | 0.091 | 1.385 | 0.168 | -0.436 | 2.483 |
| confrontation | 0.399 | 0.024 | 0.373 | 0.71 | -0.639 | 0.937 |
| Avoidance | 0.605 | 0.045 | 0.68 | 0.498 | -0.783 | 1.605 |
| Yielding | 0.594 | -0.348 | -4.404 | 0.000 | -3.791 | -1.444 |

| Multiple linear regression analysis of Functional fields in patients with hematological diseases. | | | | | | |
| --- | --- | --- | --- | --- | --- | --- |
| **Variables** | **Standard**  **error** | **Standardized regression coefficient** | **t-value** | **P-value** | **95% Confidence Interval** | |
|  |  |  |  |  | **Lower Limit** | **Upper Limit** |
| Constant | 13.981 |  | 6.426 | 0 | 62.247 | 117.452 |
| Sex | 2.637 | 0.144 | 2.425 | 0.016 | 1.189 | 11.602 |
| Age | 2.634 | 0.058 | 0.979 | 0.329 | -2.621 | 7.779 |
| Marital status | 1.618 | 0.056 | 0.935 | 0.351 | -1.681 | 4.707 |
| Place of Residence | 1.103 | -0.08 | -1.2 | 0.232 | -3.5 | 0.854 |
| Type of insurance | 2.353 | 0.03 | 0.47 | 0.639 | -3.541 | 5.75 |
| Crisis perception | 0.405 | -0.347 | -4.793 | 0.000 | -2.74 | -1.141 |
| Coping ability | 0.606 | 0.016 | 0.262 | 0.793 | -1.038 | 1.356 |
| confrontation | 0.327 | 0.026 | 0.426 | 0.67 | -0.507 | 0.786 |
| Avoidance | 0.496 | 0.011 | 0.175 | 0.861 | -0.892 | 1.066 |
| Yielding | 0.487 | -0.362 | -4.951 | 0.000 | -3.376 | -1.451 |

| Multiple linear regression analysis of Symptomatic Fields in patients with hematological diseases. | | | | | | |
| --- | --- | --- | --- | --- | --- | --- |
| **Variables** | **Standard**  **error** | **Standardized regression coefficient** | **t-value** | **P-value** | **95% Confidence Interval** | |
|  |  |  |  |  | **Lower Limit** | **Upper Limit** |
| Constant | 13.645 |  | -1.155 | 0.25 | -42.696 | 11.183 |
| Sex | 2.574 | -0.082 | -1.255 | 0.211 | -8.313 | 1.85 |
| Age | 2.571 | -0.025 | -0.375 | 0.708 | -6.039 | 4.111 |
| Marital status | 1.579 | -0.026 | -0.392 | 0.696 | -3.736 | 2.498 |
| Place of Residence | 1.076 | 0.151 | 2.065 | 0.04 | 0.098 | 4.347 |
| Type of insurance | 2.296 | -0.083 | -1.156 | 0.249 | -7.188 | 1.879 |
| Crisis perception | 0.395 | 0.218 | 2.738 | 0.007 | 0.302 | 1.862 |
| Coping ability | 0.592 | -0.082 | -1.212 | 0.227 | -1.885 | 0.451 |
| confrontation | 0.319 | 0.003 | 0.049 | 0.961 | -0.615 | 0.646 |
| Avoidance | 0.484 | 0.172 | 2.561 | 0.011 | 0.284 | 2.195 |
| Yielding | 0.476 | 0.345 | 4.285 | 0.000 | 1.099 | 2.978 |

Note：Overall QoL: R^2^ =0.365, Adj. R^2^=0.327, F=9.611, p<0.001. Functional Fields: R^2^=0.456, Adj. R^2^=0.423, F=13.975, p<0.001. Symptomatic Fields: R^2^=0.342, Adj. R^2^=0.302, F=8.664, p<0.001.
